# Stereotactic body radiotherapy for spinal oligometastases: a review on patient selection and the optimal methodology

**DOI:** 10.1007/s11604-022-01277-y

**Published:** 2022-04-09

**Authors:** Kei Ito, Yujiro Nakajima, Syuzo Ikuta

**Affiliations:** 1 Division of Radiation Oncology, Department of Radiology, Tokyo Metropolitan Cancer and Infectious Diseases Center Komagome Hospital, 3-18-22 Honkomagome, Bunkyo-ku, Tokyo, 113-8677 Japan; 2 Department of Radiological Sciences, Komazawa University, Tokyo, Japan

**Keywords:** Methodology, Oligometastases, Radiotherapy, Stereotactic body radiotherapy, Spinal metastases

## Abstract

Stereotactic body radiotherapy (SBRT) has excellent local control and low toxicity for spinal metastases and is widely performed for spinal oligometastases. However, its additional survival benefit to standard of care, including systemic therapy, is unknown because the results of large-scale randomized controlled trials regarding SBRT for oligometastases have not been reported. Consequently, the optimal patient population among those with spinal oligometastases and the optimal methodology for spine SBRT remain unclear. The present review article discusses two topics: evidence-based optimal patient selection and methodology. The following have been reported to be good prognostic factors: young age, good performance status, slow-growing disease with a long disease-free interval, minimal disease burden, and mild fluorodeoxyglucose accumulation in positron emission tomography. In addition, we proposed four measures as the optimal SBRT method for achieving excellent local control: (i) required target delineation; (ii) recommended dose fraction schedule (20 or 24 Gy in a single fraction for spinal oligometastases and 35 Gy in five fractions for lesions located near the spinal cord); (iii) optimizing dose distribution for the target; (iv) dose constraint options for the spinal cord.

## Introduction

In 1995, Hellman and Weichselbaum hypothesized that oligometastasis (≤ 5 extracranial metastases) is an intermediate state along the spectrum between local and systemic diseases [1]. According to this hypothesis, oligometastases are pathophysiologically similar to local disease and may benefit from local treatment. Some reports have suggested that it can be cured by performing local treatment with curative intent for distant metastasis (i.e., lung metastases from sarcoma, colorectal liver metastases, and extraregional lymph node metastases from cervical cancer) [2–4].

**Fig. 1:**
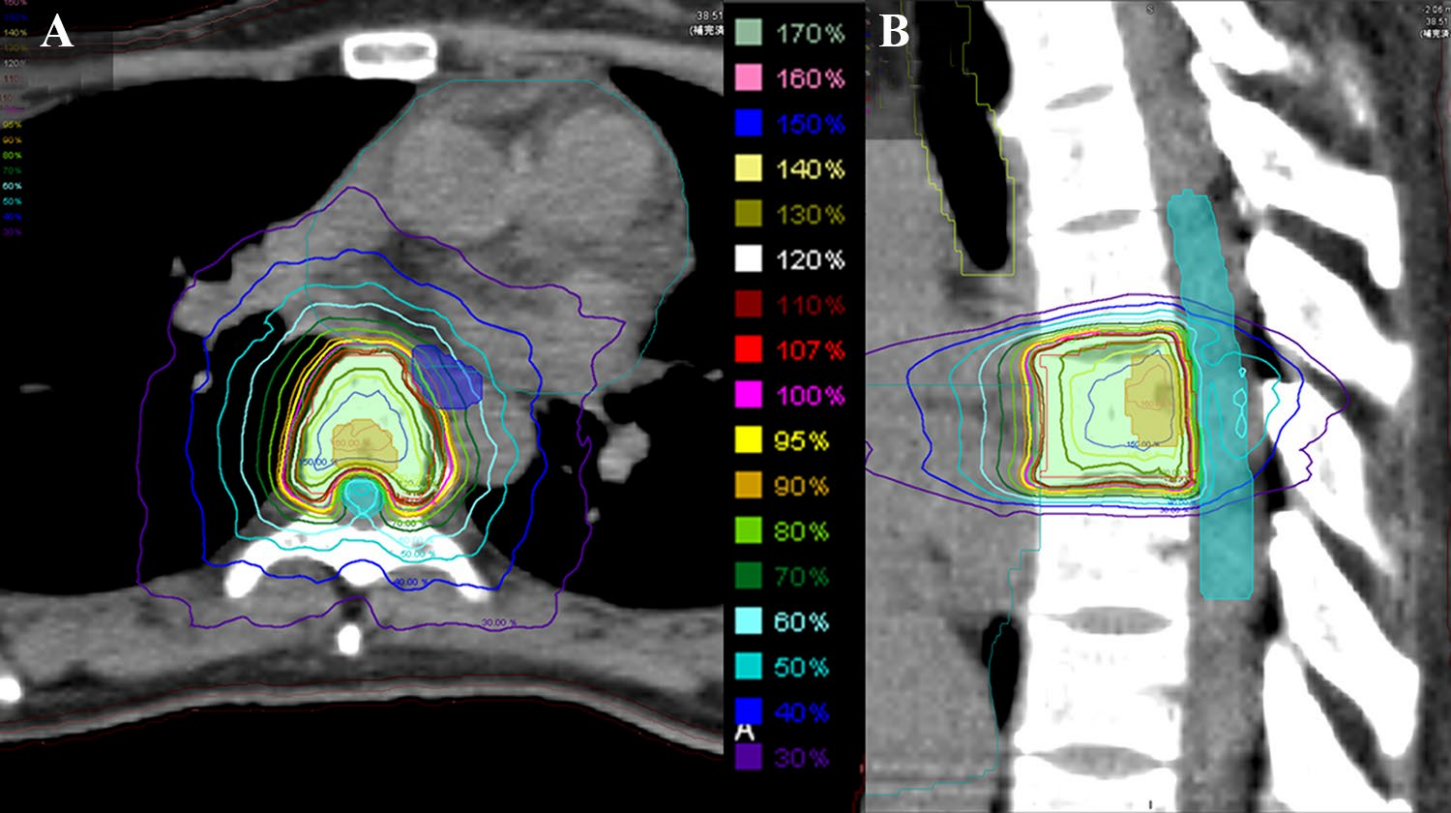
Images of a 33-year-old woman with metastatic T-6 breast cancer. **A** Axial and **B** sagittal computed tomography images with dose distribution of stereotactic body radiation therapy

Stereotactic body radiotherapy (SBRT) with intensity-modulated radiotherapy and image-guidance techniques has emerged as a new treatment option for spinal metastases (Fig. 1) [5]. Spine SBRT achieves a high local control (LC) rate (1 year: 90%) and has low toxicity (0.2% rate of neurologic injury) [6]. For spinal oligometastases, high-precision radiotherapy is essential for curative dose administration because of the positional relationship between the spinal tumor and spinal cord. Several clinical guidelines have recommended SBRT for spinal oligometastases [6, 7], and the results of the SABR-COMET trial support these recommendations [8]. The SABR-COMET trial is the first randomized study to clarify the survival benefit of SBRT for oligometastases over standard of care (median survival time: 41 vs. 28 months, *p* = 0.09). However, that trial had some limitations: it was a phase 2 trial with insufficient sample size, while more patients with prostate cancer and less than 35% of spinal metastasis cases were included in the SBRT arm [8]. In addition, none of the multiple ongoing large-scale randomized controlled trials assessing SBRT for oligometastases have reported such results (Table 1) [9–15]. Therefore, the optimal patient population among those with spinal oligometastases and the optimal methodology of spine SBRT remain unclear. The present review article considers two topics: patient selection and optimal methodology based on evidence.

**Table 1:** Large-scale randomized controlled trials assessing SBRT for oligometastases

Trial	*N*	Primary site	Number of metastases	Primary endpoint
NRG BR-002 [9]	402	Breast cancer	≤ 4	8-y OS
NRG LU-002 [10]	300	Lung cancer	≤ 3	3-y OS
SARON [12]	340	Lung cancer	≤ 3	3-y OS
CORE [12]	245	Breast, lung, and prostate cancer	≤ 3	5-y PFS
SABR-COMET 3 [13]	297	Any	≤ 3	OS
SABR-COMET 10 [14]	159	Any	4–10	OS
STEREO-STEIN [15]	280	Breast cancer	≤ 5	3-y PFS

*SBRT* stereotactic body radiotherapy, *OS* overall survival, *PFS* progression-free survival, *y* year

## Patient selection

When performing SBRT with curative intent among patients with oligometastases, it is important to select patients with particularly good prognosis and curability potential. Inappropriate patient selection for spine SBRT poses unnecessary toxicity risks to patients, such as vertebral compression fracture (VCF), esophagitis, myelopathy, and radiculopathy.

### Good prognosis

Several studies have reported on the prognostic factors after SBRT. Chao et al. generated a prognostic index based on the recursive partitioning analysis for patients treated with spine SBRT (not limited to oligometastases) [16]. Classified into three groups according to the time from primary diagnosis, Karnofsky performance status (PS), and age, the group with the best prognosis (time from primary diagnosis > 30 months and Karnofsky PS > 70) had a median survival time of 21.1 months. This result seems inadequate as a prognostic index.

Jensen et al. proposed the Prognostic Index for Spine Metastases, which was developed using data from prospective single institution trials on stereotactic spine radiosurgery [17]. The score accounts for sex, PS, previous therapy at the intended treatment site, number of organs involved, time elapsed between diagnosis and metastasis, and number of spine metastases. The scoring system categorizes patients into four groups, with a 5-year overall survival rate of 50–66% in the group with the best prognosis.

Zeng et al. compared patients who died within 3 months after spine SBRT and those who lived for > 3 years [18]. Shorter survival time after spine SBRT was observed in patients with non-breast or prostate primaries, Eastern Cooperative Oncology Group PS ≥ 2, polymetastatic disease, painful lesions, and paraspinal disease on multivariate analysis.

Although the prognostic factors of long-term survival after SBRT or surgery varied in previous studies, the identified prognostic factors tended to be related to four major overarching criteria: young age (< 65 or < 70 years), patient fitness (Karnofsky PS ≥ 70), slow-growing disease (i.e., a long disease-free interval prior to metastasis), and low disease burden (i.e., a smaller number of metastases or single-organ oligometastases) [19]. The director of the SABR-COMET trial termed these powerful prognostic factors for patients with oligometastatic disease as the “four aces” [19]. Indeed, the SABR-COMET included many patients with these characteristics (Eastern Cooperative Oncology Group PS 0–1; slow-growing disease, including prostate and breast cancer; long time from diagnosis of primary tumor to randomization; and one or two metastases), which may have been the reason for obtaining satisfactory outcomes [8].

A phase 2 trial that included 175 patients with oligometastases treated with SBRT analyzed good prognostic factors using diagnostic images [20]. They compared 5-year polymetastatic-free survival in three groups: (1) patients with a combination of < 14.8 mL oligometastatic tumor volume and a fluorodeoxyglucose (FDG) positron emission tomography (PET)-computed tomography (CT) maximum standardized uptake value (SUV_max_) < 6.5; (2) patients with a combination of < 14.8 mL oligometastatic tumor volume and an FDG-PET/CT SUV_max_ ≥ 6.5; (3) patients with an oligometastatic tumor volume ≥ 14.8 mL regardless of the SUV_max_. The 5-year polymetastatic-free survival rates were 89%, 58% (*p* = 0.02), and 17% (p < 0.001) in Groups 1, 2, and 3, respectively. These findings suggest that the total gross tumor volume (GTV) of the oligometastases and the SUV_max_ are useful in determining whether the patients have a potential risk of polymetastases.

### Use of advanced diagnostic images

Advanced diagnostic imaging modalities are effective in accurately diagnosing a small number of metastases and excluding polymetastases. FDG-PET/CT and diffusion-weighted whole-body imaging with background signal suppression (DWIBS) magnetic resonance imaging are useful for diagnosing oligometastases because they are capable of whole-body evaluation and have the spatial resolution necessary to identify targets for SBRT [21]. In addition, these tests have higher sensitivity and specificity than bone scintigraphy [22]. Although PET-CT has the advantage of easily identifying lesions in the acquired images, its limitation lies in radionuclide accumulation in regions of high glucose metabolism, such as areas with non-specific inflammation and physiological changes other than tumors. For osteoblastic metastases, PET is less suitable than bone scintigraphy, and confirmation of osteosclerosis on CT is desirable [22]. DWIBS reflects the motion restriction of water molecules in areas of high cell density and is another tool for detecting metastasis [23]. The main advantage of DWIBS is that it is less invasive, as there is no exposure to radiation and images can be acquired without the use of contrast media [24]. However, lesions in bones with high signal intensity on T2-weighted images, such as hemangiomas or red bone marrow in patients with anemia, can be judged as abnormal signals. Such images require great skill for accurate interpretation [25, 26]. Previous reports assessing the use of PET-CT and DWIBS to detect bone metastases have shown that they have similar sensitivities; however, the specificity of DWIBS may be lower than that of PET-CT [27–29]. In addition, prostate-specific membrane antigen-PET is suitable for assessing bone metastasis in prostate cancer if the above tests do not provide satisfactory results [30].

## Optimal methodology

The purposes of spine SBRT are as follows: complete control of oligometastasis, relief of painful lesions, and improvement of neurologic function for patients with epidural spinal cord compression [31]. Among these, SBRT for oligometastases must be an extremely aggressive treatment strategy and achieve a high LC rate because of its curative intent.

A previous report suggested the importance of SBRT methodologies for oligometastases. A phase 3 randomized trial comparing SBRT doses for oligometastases (more than 90% of patients had bone metastases) showed that high-dose SBRT (24 Gy) in a single fraction had a significantly lower local failure rate than medium-dose SBRT (27 Gy) in three fractions [32]. Notably, the cumulative incidence of distant metastatic progression was also significantly lower in the high-dose SBRT group (3 years: 5.3% vs. 22.5%, *p* = 0.01). Therefore, appropriate local treatment for the initial metastases may prevent progression to systemic disease. Herein, we suggested countermeasures to improve LC in patients with spinal oligometastatic disease receiving SBRT.

### Delineation of the target

In the early days of spine SBRT, no clinical target volume (CTV) margin was popular [33–35]. However, a retrospective study showed that contouring the whole vertebral body tended to improve LC compared to contouring part of the vertebral body [36]. In addition, the International Spine Radiosurgery Consortium recommends CTV expansion based on the GTV location, wherein the spine is divided into six sectors (the vertebra, both pedicle, both transverse process, and spinous process) and the CTV includes the sectors where the GTV is located and the sectors adjacent to the GTV for subclinical tumor spread in the marrow space [37]. This standard setting should be enforced to achieve a high LC rate.

### Dose fraction schedule

Various dose fractionations are used depending on the facility, and there is no broad consensus on the optimal dose fractionation in SBRT for spinal oligometastases [38]. A meta-analysis comparing the prescribed dose of spine SBRT showed that a higher cumulative dose led to a higher 2-year LC rate in patients receiving 1–5 fractions [39]. Dose fraction schedules should be selected from those used in the randomized controlled trials of the SABR-COMET trial and the dose comparison trial conducted by Zelefsky et al. (Table 2) [8, 32]. If the treatment goal is a 2-year LC rate > 80%, appropriate dose fractionations are 18 Gy/1 Fr, 20 Gy/1 Fr, 24 Gy/1 Fr, 30 Gy/3 Fr, and 35 Gy/5 Fr. The regimen with the highest LC rate is 24 Gy in a single fraction (estimated 2-year LC of 96%) [39].

**Table 2:** Local control rate and dose fraction schedules used in randomized trials [39]

Dose fractionation	2-y LC rate (%)
16 Gy/1 Fr [8]	72
27 Gy/3 Fr [32]	78
18 Gy/1 Fr [8]	82
35 Gy/5 Fr [8]	83
30 Gy/3 Fr [32]	85
20 Gy/1 Fr [8]	90
24 Gy/1 Fr [32]	96

*LC* local control, *y* year, *Fr* fraction

In cases wherein the tumor is adjacent to the spinal cord [minimum distance between the GTV and planning organ-at-risk volume (PRV) of the cord < 3 mm [40]], we may have to increase the number of the fraction size of SBRT. A dose constraint of the spinal cord for the radiation-naive region is 12.4 Gy in a single fraction [41], resulting in a delivery of 27.78 Gy [biological equivalent dose with α/β = 10 (BED10)] to the epidural tumor in contact with the spinal cord. In a regimen involving five fractions, as the spinal cord dose constraint would be 25.3 Gy [41], 38.10 Gy (BED10) can be administered to the epidural tumors. By increasing fractionation, it is possible to escalate the minimum dose delivered to a gross tumor, which would contribute toward achieving LC [42–44]. However, the benefit of increasing fractionation may be limited or none in cancer types with low α/β such as breast and prostate cancers. It is noted that for high-grade epidural spinal cord compression, surgical intervention should precede SBRT to deliver a sufficiently high tumoricidal dose [45].

VCF is a relatively common adverse effect of spine SBRT. Several studies have identified that high dose per fraction is a risk factor for VCF development [38, 46]. A multi-institutional retrospective study showed that the cumulative incidence of VCF at 1 year was 39% with ≥ 24 Gy/fraction, 19% with 20–23 Gy/fraction, and 10% with ≤ 19 Gy/fraction [46]. Although increasing the number of fractions and reducing the dose per fraction are effective in avoiding VCF, it should be recognized that VCF is an adverse effect that is painless in most cases [47] and does not directly correlate with survival.

From the aforementioned studies, the recommended doses are 20 or 24 Gy in a single fraction for spinal oligometastases and 35 Gy in five fractions for lesions close to the spinal cord (minimum distance between GTV and PRV of the cord < 3 mm).

### Optimizing dose distribution for the target

The prescribed dose is generally delivered up to 90–95% of the planning target volume (PTV) [8, 48]. In addition, the isodose prescription is used to sharply reduce the dose outside the PTV while administering a high dose within the PTV. Several dosimetric analyses have shown a positive correlation between LC and the marginal dose to the GTV but not the dose to the PTV [42–44]. Retrospective data from the MD Anderson Cancer Center showed that patients with a minimum dose to the GTV ≥ 14 Gy in a single fraction had a significantly higher LC rate than those with a minimum dose < 14 Gy [42]. In addition, retrospective data from the Memorial Sloan-Kettering Cancer Center suggested that the thresholds of local failure were a minimum dose to the GTV of < 15 Gy [43] and a dose that covered 95% of the GTV ≤ 18.3 Gy in a single fraction [44]. Therefore, the isodose prescription, which produces a steep dose gradient and a hotspot in the target, is valid concerning dose reduction to the organs at risk and excellent LC.

Optimizing the treatment plan increases the target dose coverage and reduces the inter- and intra-planner variability [41]. If the GTV is not located near the spinal cord, the GTV dose can be increased by setting a large limit on the maximum dose in the target compared to the prescribed dose (e.g., the protocol of CCTG SC.24/TROG 17.06 allows a maximum dose of + 50% in the PTV [47]). If the GTV is in contact with the spinal cord, the minimum GTV dose should be as close as possible to the spinal cord dose constraint (prioritize the dose constraint). The target dose coverage should be optimized based on past clinical trial protocols [48], with the goal of achieving at least 90–95% coverage of the PTV by the prescribed dose. The created dose distribution should be visually checked for a steep dose gradient around the spinal cord, considering that the maximum photon dose fall-off gradient is approximately 10–13% per millimeter [40].

### Alleviation of the dose constraint for the spinal cord

Table 3 summarizes the representative dose constraints for the spinal cord in de novo SBRT [49–54]. There are three dose constraints of 12.4, 14, and 16 Gy in a single fraction at the maximum point dose (with point defined as ≤ 0.035 cc [50]). Several studies examining SBRT for de novo spinal metastases using the strictest constraint (maximum point dose of 17 Gy in two fractions for thecal sac or the PRV of the cord [49]) have not observed radiation myelopathy [55–57]. Reports that calculated dose constraint of 14 Gy have adopted the spinal cord itself (without PRV margin) as the structure of interest [50–53] (Table 3). Some reports that used this setting have not confirmed radiation myelopathy in the long-term follow-up examination [48, 58]. Alleviating the dose constraint of the spinal cord is an option for increasing the minimum dose delivered to an epidural tumor. However, radiation oncologists should use the 16 Gy dose constraint with caution in clinical practice because of the small sample size of this phase 1 trial [54].

**Table 3:** Representative dose constraints for the spinal cord (maximum point dose)

	Dose reporting structure	1 Fr	2 Fr	3 Fr	5 Fr
Sahgal et al. [49]	Thecal sac	12.4 Gy	17 Gy	20.3 Gy	25.3 Gy
AAPM TG101 [50]	Spinal cord	14 Gy	N/A	21.9 Gy	30 Gy
Kim et al. [51]	Spinal cord and medulla	14 Gy	18.3 Gy	22.5 Gy	28 Gy
Katsoulakis–Gibbs model [52, 53]	Spinal cord	14 Gy	19.3 Gy	23.1 Gy	28.8 Gy
Ghia et al. [54]	Spinal cord	16 Gy	N/A	N/A	N/A

*AAPM* The American Association of Physicists in Medicine, *TG* task group, *N/A* not available, *y* year, *Fr* fraction

## Conclusion

Although SBRT can cure some patients with oligometastases, the optimal patient population and methodology for spine SBRT remain unknown. Patients with young age, good fitness, slow-growing disease, low disease burden, and mild FDG accumulation in PET are suitable for spine SBRT with curative intent. In addition, the use of appropriate diagnostic imaging can exclude the possibility of false oligometastases. Regarding the optimal methodology, we proposed four countermeasures to improve LC. We believe that this information will be useful when selecting patients and performing appropriate spine SBRT in daily clinical practice.
